# Anti-COVID-19 Vaccination in Patients with Autoimmune-Autoinflammatory Disorders and Primary/Secondary Immunodeficiencies: The Position of the Task Force on Behalf of the Italian Immunological Societies

**DOI:** 10.3390/biomedicines9091163

**Published:** 2021-09-04

**Authors:** Raffaele D’Amelio, Riccardo Asero, Marco Antonio Cassatella, Bruno Laganà, Claudio Lunardi, Paola Migliorini, Roberto Nisini, Paola Parronchi, Isabella Quinti, Vito Racanelli, Gianenrico Senna, Angelo Vacca, Enrico Maggi

**Affiliations:** 1 Dipartimento di Medicina Clinica e Molecolare, Sapienza Università di Roma, Via di Grottarossa 1035-1039, 00189 Rome, Italy; raffaele.damelio@uniroma1.it; 2 Ambulatorio di Allergologia, Clinica S. Carlo di Paderno Dugnano, Via Ospedale 21, 20037 Milano, Italy; r.asero@libero.it; 3 Sezione di Patologia Generale, Dipartimento di Medicina, Università di Verona, Strada Le Grazie 4, 37134 Verona, Italy; marco.cassatella@univr.it; 4 UOC Medicina Interna, Dipartimento di Medicina Clinica e Molecolare, AOU S. Andrea, Sapienza Università di Roma, Via di Grottarossa 1035-1039, 00189 Rome, Italy; bruno.lagana@ospedalesantandrea.it; 5 Responsabile Unità di Malattie Autoimmunitarie, Dipartimento di Medicina, AOU Policlinico G.B. Rossi, Borgo Roma, Università di Verona, Piazzale Ludovico Antonio Scuro 10, 37134 Verona, Italy; claudio.lunardi@univr.it; 6 Direttore Unità Operativa di Immunoallergologia Clinica, Dipartimento di Medicina Clinica e Sperimentale, Azienda Ospedaliero Universitaria Pisana, Università di Pisa, Via Roma 67, 56126 Pisa, Italy; paola.migliorini@med.unipi.it; 7 Direttore Reparto Immunologia, Dipartimento di Malattie Infettive, Istituto Superiore di Sanità, Viale Regina Elena 299, 00161 Rome, Italy; roberto.nisini@iss.it; 8 Direttore SOD Immunologia e Terapie Cellulari, Dipartimento di Medicina Sperimentale e Clinica, AOU Careggi, Università di Firenze, Largo Brambilla 3, 50134 Firenze, Italy; paola.parronchi@unifi.it; 9 Responsabile UOD Centro di Riferimento Regionale per le Immunodeficienze, Dipartimento di Medicina Molecolare, AOU Policlinico Umberto I, Sapienza Università di Roma, Viale dell’Università 37, 00161 Rome, Italy; isabella.quinti@uniroma1.it; 10 UOC Medicina Interna “Guido Baccelli”, Dipartimento di Scienze Biomediche ed Oncologia Umana, AOU Policlinico, Università di Bari, Piazza Giulio Cesare 11, 70124 Bari, Italy; vito.racanelli1@uniba.it; 11 Direttore USD Allergologia, Dipartimento di Medicina, AOU Policlinico G.B. Rossi, Borgo Roma, Università di Verona, Piazzale Ludovico Antonio Scuro 10, 37134 Verona, Italy; gianenrico.senna@aovr.veneto.it; 12 Direttore UOC Medicina Interna “Guido Baccelli”, Dipartimento di Scienze Biomediche ed Oncologia Umana, AOU Policlinico, Università di Bari, Piazza Giulio Cesare 11, 70124 Bari, Italy; angelo.vacca@uniba.it; 13 Unità di Immunità Traslazionale, Dipartimento di Immunologia, Ospedale Pediatrico Bambino Gesù, IRCCS, Viale di S. Paolo 15, 00146 Rome, Italy

**Keywords:** COVID-19, SARS-CoV-2, vaccines, auto-immune auto-inflammatory disorders, primary immunodeficiencies, secondary immunodeficiencies

## Abstract

The Coronavirus disease 2019 (COVID-19) pandemic has represented an unprecedented challenge for humankind from health, economic, and social viewpoints. In February 2020, Italy was the first western country to be deeply hit by the pandemic and suffered the highest case/fatality rate among western countries. Brand new anti-COVID-19 vaccines have been developed and made available in <1-year from the viral sequence publication. Patients with compromised immune systems, such as autoimmune-autoinflammatory disorders (AIAIDs), primary (PIDs) and secondary (SIDs) immunodeficiencies, have received careful attention for a long time regarding their capacity to safely respond to traditional vaccines. The Italian Immunological Societies, therefore, have promptly faced the issues of safety, immunogenicity, and efficacy/effectiveness of the innovative COVID-19 vaccines, as well as priority to vaccine access, in patients with AIADs, PIDs, and SIDs, by organizing an ad-hoc Task Force. Patients with AIADs, PIDs, and SIDs: (1) Do not present contraindications to COVID-19 vaccines if a mRNA vaccine is used and administered in a stabilized disease phase without active infection. (2) Should usually not discontinue immunosuppressive therapy, which may be modulated depending on the patient’s clinical condition. (3) When eligible, should have a priority access to vaccination. In fact, immunizing these patients may have relevant social/health consequences, since these patients, if infected, may develop chronic infection, which prolongs viral spread and facilitates the emergence of viral variants.

## 1. Introduction

The Coronavirus disease 2019 (COVID-19) pandemic as of 10 July 2021 has globally caused 186,152,198 cases and 4,021,065 deaths, whereas 3,393,833,500 anti-COVID-19 vaccine doses have been administered (https://coronavirus.jhu.edu/map.html, accessed on 22 July 2021). In February 2020, Italy was the first western country to be deeply hit by the pandemic, which developed in at least three consecutive waves, the first in the first half of 2020, the second and the third between the last trimester of 2020 and the first half of 2021. One characteristic of Italy has been, since the beginning, a high case/fatality rate. In fact, according to Johns Hopkins University, Italy at the global level ranks 10th for number of cases, after USA, India, Brazil, France, Russia, Turkey, UK, Argentina, and Colombia, but among these countries, Italy shows the highest case/fatality rate of 2.99%. The high number of cases and the high lethality, the first among western countries, prompted the three Italian Immunological Societies (*Associazione Allergologi ed Immunologi Italiani Territoriali ed Ospedalieri* [AAIITO], *Società Italiana di Allergologia, Asma ed Immunologia Clinica* [SIAAIC], *Società Italiana di Immunologia, Immunologia Clinica e Allergologia* [SIICA]), as soon as anti-COVID-19 vaccines became available, to set up a Task Force, including immunologists and clinical immunologists, to draw up guidelines on COVID-19 vaccination in vulnerable patients with diseases of the immune system, such as autoimmune-autoinflammatory disorders (AIAIDs), as well as primary (PIDs) and secondary (SIDs) immune deficiency diseases. In particular, the Task Force has faced the following issues: (1) analyzing the different anti-COVID-19 vaccine types in order to identify possible contraindications of specific vaccines in these patients; (2) establishing the compatibility of the different diseases of the immune system with these innovative vaccines; (3) evaluating the possible interference of the different therapies with the vaccines and the consequent possible interruption or modification of the doses of these therapies; (4) identifying criteria to fix priority levels in the access to vaccination, based on the major risk of infection and disease severity.

Vaccinations are a safe and effective tool for prevention and control of infectious diseases, particularly in patients with AIAIDs, PIDs, and SIDs. In fact, these patients have a higher infection risk, thus safety, immunogenicity, and efficacy of traditional vaccines, as well as possible vaccine contraindications, has been widely and thoroughly studied [1,2,3,4,5,6,7,8,9,10,11,12,13,14,15] (Table 1, Table 2 and Table 3) Therefore, the Task Force adopted the method of carefully analyzing the literature, to examine the response of the different pathologies of the immune system to the traditional vaccines, as the basis for inferring possible reactions of these pathologies challenged with the innovative anti-COVID-19 vaccines [1,2,3,4,5,6,7,8,9,10,11,12,13,14,15] Table 1, Table 2 and Table 3 The Task Force worked by teleconference for a total of six meetings during the period of March–May 2021.

## 2. COVID-19 Vaccines

The anti-COVID-19 vaccine development has resulted in an unprecedented global effort, which has allowed the first vaccine to be approved for human use less than one year after the publication of the SARS-CoV-2 spike protein-coding sequence on 10 January 2020 [16]. As of 12 June 2021, 287 COVID-19 vaccines are under development, 102 of which are in clinical trials (32 protein subunits, 16 viral vector non-replicating, 16 inactivated viruses, 16 messenger RNA (mRNA), 10 DNA, 5 virus-like particles, 2 viral vectors replicating, 2 viral vector replicating + antigen presenting cells, 2 live attenuated viruses and 1 viral vector non-replicating + antigen presenting cell), and the remaining 185 are in the pre-clinical phase. Of the former 102, six are already approved for human use (two mRNA vaccines, three viral vector non-replicating vaccines and one inactivated vaccine, Table 2), whereas another 15 are in phase 3 of study, including six inactivated virus vaccines, five protein subunits, two mRNA, one DNA and one viral vector non-replicating. The latter, corresponding to the Russian vaccine from Gamaleya Research Institute, has been already used in humans for many months, as approved by the Russian Ministry of Health, but is still reported in the WHO document as phase 3 [17]. The same is true for BBV152 inactivated vaccine, from Bharat Biotech, which has been approved and used in India [18].

Anti-COVID-19 vaccines of the traditional type that are composed of inactivated virus or recombinant Spike protein and adjuvant [19], for which wider scientific knowledge and clinical experience are available, could preferentially be indicated in patients with AIAIDs, PIDs, and SIDs. However, despite demonstrated efficacy for the NVX-CoV2373 of 89.7% [20], no traditional vaccines are approved by the European Medicines Agency (EMA) yet (Table 4.)

Anti-COVID-19 vaccines based on non-replicating viral vectors, such as those from AstraZeneca (Vaxzevria) [21,22], Janssen/Johnson & Johnson [23,24], Cansino Biological Inc. [25], and Gamaleya Research Institute [26,27] (Table 4.). are being used for mass vaccination (only the first two vaccines are currently used in Italy). They harness the technology already applied to the anti-Ebola vaccine. AstraZeneca uses an adenovirus from the chimpanzee as a viral vector, Janssen/Johnson & Johnson uses the human adenovirus 26, Cansino Biological Inc. uses the human adenovirus 5, whereas Gamaleya uses two recombinant human adenoviruses: adenovirus 26 for the first dose and adenovirus 5 for the second dose. One concern of these types of vaccines is the previous and effective anti-viral vector immune response, which may fully inactivate the vaccine; however, this problem involves the general population, not only patients with AIAIDs, PIDs, and SIDs, and may be reduced by using viral vectors from primates. The post-vaccine protection is complete 1–2 weeks following the end of vaccination schedule. In the case of Vaxzevria, protection from severe diseases has been calculated at approximately 70% after the second of two doses, whereas for the Janssen/Johnson & Johnson vaccine it has been observed that vaccinated people were protected at 73% and 82% at 14 and 28 days, respectively, from the single dose administration [24]. During the registration studies, substantial safety of these vaccines was observed [21]. However, patients with AIAIDs, PIDs, and SIDs were not enrolled in these phase 3 studies and therefore it is not possible to draw definitive conclusions for the safety and efficacy in these patients.

**Table 4:** Characteristics of anti-COVID-19 vaccines available or in advanced phase of approval.

Vaccines	Composition	Cellular Immunity	Neutralizing Antibodies	Doses	Efficacy	References
Pfizer Comirnaty	30 µg mRNA Spike	Yes	Yes	2, 3 weeks apart	95%	[28]
Moderna Spikevax	100 µg mRNA Spike	Yes	Yes	2, 4 weeks apart	94.1%	[29]
AstraZeneca Vaxzevria	Non-replicating viral vector Spike DNA	Yes	Yes	2, 4–12 weeks apart	70%	[21,22]
Janssen (J&J)	Non-replicating viral vector Spike DNA	Yes	Yes	1	73–82% at 14–28 days, respectively	[23,24]
Cansino Biological Inc.	Non-replicating viral vector Spike DNA		Yes	1		[25]
Sinovac CoronaVac	Inactivated whole virus		Yes	2, 2 weeks apart	83.5%	[30,31]
Gamaleya Res. Institute	Non-replicating viral vector Spike DNA		Yes	2, 3 weeks apart	91.6%	[26,27]
BBV152 Bharat Biotech	Inactivated whole virus		Yes	2, 2 weeks apart		[32,33]
Novavax	Recombinant Spike+ Adjuvant	Yes	Yes	2, 3 weeks apart	89.7%	[19,20]

A very rare, severe, unpredictable, and frequently lethal thrombosis in uncommon sites, such as the cerebral venous sinus and the splanchnic venous circulation, has emerged in post-approval vaccine safety surveillance after large-scale vaccination. It is characterized by thrombocytopenia and anti-PF4 IgG antibodies and has been associated with administration of non-replicating viral vector vaccines (AstraZeneca and Janssen/Johnson & Johnson vaccines). Its pathology shares some characteristics with the heparin-induced thrombocytopenia [34] and has been named “vaccine-induced immune thrombotic thrombocytopenia” (VITT) [35]. Lethality is approximately 50% in Europe with AstraZeneca and approximately 25% in the USA with Janssen/Johnson & Johnson [36], especially in women < 60 years old. The alarm caused by the deaths of healthy young people, mainly women, induced the Italian Medicines Agency (AIFA) to recommend the AstraZeneca vaccine only for subjects > 60 years old, considering the higher risk of VITT in younger people, who, however, have a lower risk of getting infected with a severe form of COVID-19. Moreover, the hesitancy of the population towards Vaxzevria pushed the health authorities in many countries to consider the possibility of a heterologous prime boost in people who had received the first dose of Vaxzevria, although the risk of getting VITT following the second dose is even substantially lower than the already low risk associated with the first dose. The heterologous prime-boost vaccine policy is successful with other vaccines [37], such as polio [38]. This seems to be the case even for COVID-19, as shown at experimental [39,40] and clinical levels by the Spanish study CombiVacS, carried out on over 660 subjects already vaccinated with the first Vaxzevria dose, who received a second dose of a Pfizer mRNA vaccine not before eight weeks following the first dose [41]. This heterologous approach, in analogy with the observation in experimental animals [40], appears highly effective also in humans, with immune responses even higher than those observed after two mRNA vaccine doses. Recently, in 88 health care workers who had received a first dose of Vaxzevria, 37 chose a homologous and 51 a heterologous booster with mRNA-1273 (Moderna). Seven–ten days following the second dose, those who had received the homologous booster had a five-fold increase of specific anti-Spike and anti-receptor binding domain (RBD) of severe acute respiratory syndrome coronavirus 2 (SARS-CoV-2) antibodies compared with the pre-booster levels, whereas those who chose the heterologous booster had a 115-fold and 125-fold increase of anti-Spike and anti-RBD antibodies, respectively [42]. However, a preliminary study on 830 subjects, in whom the adverse effects (AEs) were analyzed following either two doses of the same vaccine, Pfizer or AstraZeneca, or two doses of different vaccine types, the first Pfizer and the second AstraZeneca, or vice versa, showed a higher prevalence of AEs in the group receiving different vaccine types than the group receiving the same type [43]. The higher prevalence of AEs in patients receiving heterologous vaccine doses was not confirmed in two smaller studies, the first on 326 healthcare workers [44] and the other on 26 subjects [45]. In the latter study, cellular and humoral immunogenicity were explored and the results confirmed the data on the high stimulation of the immune system by the heterologous immunization already observed in the Spanish study, even towards the variants of concern. This vaccine strategy has been adopted in Italy and in some other European countries. It may be adopted not only for the second vaccine dose, but even for the possible follow-up doses, which may become necessary in consequence of the emergence of new viral variants poorly addressed by the current vaccines and in the case of passage of the pandemic to an endemic state, similar to influenza.

Anti-COVID-19 vaccines based on the mRNA technology, such as those developed by Pfizer/Biontech (Comirnaty) [28] and Moderna (Spikevax) [29], have never been used before in large-scale vaccination programs. mRNA coding for the Spike protein of SARS-CoV-2 is delivered in lipid nanoparticles to favor its entry into the cell and to protect it from circulating and tissue RNAses [46,47]. This new category of vaccines, composed of nucleic acids and liposomes, is administered without adjuvants or vectors, considering that mRNA behaves as an adjuvant by interacting with intracellular (endosomal) Toll-like receptors (TLRs), thus activating the inflammation networks [46]. Published data on the phase 3 trials of the two already authorized vaccines refer to a non-infected population of over 43,000 and 30,000 subjects, respectively, treated with two vaccine doses (each of 30 and 100 µg, respectively) or placebo. After four months of observation, the groups treated with the two vaccines showed an efficacy, which is a reduction of disease cases (effectiveness has the same meaning, but it is used in real-life conditions), of 95% and 94.1%, respectively, compared to the group of subjects who had received placebo, and the safety was considered optimal (Table 4) [28,29]. These similar results are very important, as they have been obtained on two different populations, immunized with different, but very similar, vaccines produced with the same technology. Although in the pre-approval studies details on the possible presence of patients with AIAIDs in the study population were not provided, recently a study reporting 325 patients with rheumatic diseases who had received the first dose of an mRNA vaccine (51% Pfizer and 49% Moderna) was published. Observed AEs were no more frequent and/or serious than those reported in the general population and disease flare-ups were not described in vaccinated patients [48]. The same results were obtained in two recent smaller studies, including 26 and 70 patients, respectively [49,50]. The mRNA vaccines are generally well tolerated, with transient local and systemic AEs. In the first phase of large-scale immunization, mainly in the USA, the most serious AE was represented by the anaphylactic shock from sensitization to polyethylene-glycol 2000, which occurred rarely but was approximately 10-fold more frequent than with traditional vaccines and often in non-allergic people [47]. Following Pfizer and Moderna vaccines, 20 cases of thrombocytopenia were recently described in the USA [51]. In 17 subjects, pre-vaccine thrombocytopenia was not present. Although the relationship with the vaccine has not been definitively demonstrated, in 19 cases thrombocytopenia appeared after the first vaccine dose. However, the severity was milder and the prognosis favorable compared to VITT. Recently, in Israel and the USA, a very rare (1/100,000 and 0.52/100,000, respectively) post-mRNA vaccine myocarditis, mainly occurring in <30-year-old men after the second dose, has been described [52]. A retrospective study on 23 post-vaccine cases of myocarditis occurring in the US military suggests that the observed cases were higher than expected and that in 16/23 cases cardiac symptoms resolved within one week, whereas in the other seven cases, symptoms continued to the time of the publication of the study [30].

The inactivated traditional vaccine from Sinovac seems to be well tolerated and quite immunogenic [31], even in children and adolescents 3–17 years old, as observed in a phase 1/2 study [32], and an efficacy of 83.5% was just reported [33]. Another inactivated vaccine with an adjuvant, BBV152, is approved in India [18]. Its safety and immunogenicity seem satisfactory, whereas poor information is available on adaptive cellular immunity [53] and efficacy, considering that a phase 3 study has not yet been published. The vaccine-induced protection appears complete in rhesus macaques [54].

Regarding the fear that the currently approved vaccines may fail to mitigate or prevent infection with the viral variants of concern, the analyses carried out on the sera of the vaccinated subjects indicate that the immune response may be unmodified or reduced, whereas for all these variants increased transmissibility [55,56,57,58] and disease severity [59,60,61,62,63] have been identified (Table 5) [64]. However, published data show a markedly reduced neutralizing capacity towards the South-African B.1.351 (now denominated beta) variant of 2/3 for the Pfizer vaccine [65] and of 1/6 for the Moderna vaccine [66]. A marked reduction has even been reported for the AstraZeneca vaccine [56]; for this reason, its trial in Africa was cancelled. It must be underlined that up to now a clear identification of the threshold for protection of neutralizing antibodies is lacking. Moreover, the adaptive cellular immunity may replace the lack of protective antibodies [67]. Thus, it is still premature to assert that the virus variants, in particular the South-African one, are not covered by the currently approved vaccines; moreover, not-yet-approved vaccines seem to be more active against the variants [68,69]. As a further confirmation, there is the recently published use of the Pfizer vaccine in Qatar, where 50% of the COVID-19 cases are due to the South-African variant and 44.5% to the B.1.1.7. (so-called English, currently denominated alpha) variant; the vaccine-induced protection against infection by the English variant was 89.5% and by the South-African variant was 75% two weeks after the second dose, but 100% towards severe disease by the two variants [70]. The sera of subjects vaccinated with two doses of Comirnaty or Vaxzevria and collected between one and four weeks from the second dose showed a significant 2.6-fold and 2.9-fold reduction, respectively, of neutralizing activity against the P.1 Brazilian variant (currently denominated gamma) compared to the Victoria viral strain [71]. However, on the basis of epidemiological data showing a possible prevalent circulation of new variants of concern, which may be poorly recognized by the current vaccines (this seemed to be the case for B.1.617.2, the so-called Indian variant, currently denominated delta, even though preliminary data show an effectiveness of 79% for Comirnaty and 60% for Vaxzevria [69], which was recently reconfirmed as 88% and 67%, respectively [72]), it is assumed that the administration of a further dose of vaccine will occur that is adequately modified to intercept the new prevalent variants. In fact, the currently approved vaccines are easily and quickly modifiable in order to be tailored to the prevalent variants (Pfizer-Biontech Press release, 8 July 2021). Considering that the RNA viruses have a marked trend to mutate, the possibility that the general population will be forced to be vaccinated every year towards the prevalent circulating variant, as with influenza, is a likely scenario. However, for the general population, similarly to influenza, annual boosters might be envisaged, and it is possible that additional doses should be considered for vulnerable immunosuppressed patients, mounting an inadequate protective immune response after a conventional cycle of vaccination, as observed in transplanted patients [73], probably in relation to the treatment with mycophenolate and glucocorticoids [74]. Indeed, in over 100 transplanted patients, 44% reached a meaningful antibody production only following a third vaccine dose [75], and one patient with AIAD needed to receive four doses to develop seropositivity [76].

Neutralizing antibodies against the original viral strain from Wuhan were always present in the sera from subjects who recovered from COVID-19, whereas they were identified only sporadically against the B.1.351 variant. However, following one booster with an mRNA vaccine, the neutralizing capacity, even towards the variants, increased one thousand-fold, whereas a second dose did not have further effect [77]. These data have been recently confirmed [78,79], thus providing a solid scientific basis for the recommendation of administering a single vaccine dose in subjects who have recovered from COVID-19 infection.

Considering that the vaccines are innovative and have been authorized and used for only a few months, nothing is known about the possible appearance of long-term AEs after vaccination and the duration of immune response at protective levels. In fact, registration studies have only been observed during a four-month period. However, it is known that humoral immunity towards SARS-CoV-1 disappears after two–three years, whereas adaptive cellular immunity persists for a longer time [80,81]. Recently, a study was published showing that post-infection anti-COVID-19 neutralizing antibodies persist at protective levels up to eight months [82], and probably even more [78].

A further relevant issue is the type of vaccine-induced protection, whether addressed to prevent infection or only disease severity. The vaccine-induced protection observed in experimental models and in humans in phase 3 studies has not provided evidence that the vaccine prevents the possibility of infection [22,24,28,29,83,84,85]. However, the recent identification of salivary IgA in subjects vaccinated with two doses of mRNA vaccines [86] is reassuring because, even though its protective role has not been explored, it allows us to rule out the fear of a poor IgA mucosal immune response [87].

Pregnancy is listed as a contraindication on the labels of the Pfizer and Moderna vaccines, which the Food and Drug Administration (FDA) approved for emergency use. However, a critical analysis of this point is ongoing. EMA seems inclined to remove the warning. A preliminary study does not show relevant safety concerns [88] and even immunogenicity seems adequate [89], with the appearance of vaccine-induced antibodies which pass through the placenta and into milk. In fact, breastfeeding is not contraindicated. Recent data show a good safety and immunogenicity profile. Two weeks following vaccination, secretory IgA is already present in the maternal milk and remains, together with IgG, for approximately six weeks [90]. The pivotal role of secretory IgA in the SARS-CoV-2 neutralization has been recently underlined [91].

The mRNA vaccines have been approved for individuals older than 16 years (Pfizer) and 18 years (Moderna). However, recently the EMA has even approved Comirnaty Pfizer for administration to adolescents in the age range of 12–16 years, based on a study showing an excellent immune response in this age range [92]. Spikevax Moderna has filed for emergency approval of its COVID-19 vaccine for teens and the EMA has recently recommended granting an extension of its use to ages 12–17 years.

## 3. Safety of COVID-19 Vaccines in Patients with AIAIDs, PIDs, and SIDs

The issue of safety of the COVID-19 vaccines has been based on what is already known for traditional vaccines (Table 1, Table 2 and Table 3) [1,2,3,4,5,6,7,8,9,10,11,12,13,14,15]. In general, patients with AIAIDs, PIDs, and SIDs may safely receive inactivated or subunit vaccines, whereas the living vaccines should generally be avoided. Moreover, in patients with AIAIDs the issue of a possible flare-up of a stabilized disease because of vaccine stimulation should be considered. This underlines the compatibility of adjuvanted vaccines with patients with AIAIDs: in fact, adjuvants may be useful in immunocompromised patients, but the hyper-stimulation of a deranged immune system may run uncontrolled. The COVID-19 innovative mRNA vaccines behave like an adjuvant, and this should carefully be considered in some AIAIDs. It must be underlined that in the pre-approval studies the observation period was relatively short. Moreover, in the study population of the pre-approval studies, no patients with AIAIDs or PIDs were included. Only a small percentage of patients with SIDs were enrolled, and their clinical data has not been reported. Further studies, including a larger sample of these patients, treated with different immunosuppressive drugs, and analyzed during a longer period, are mandatory. Despite the current lack of definitive recommendations, some preliminary studies [48,49,50], supported by a recent larger multicenter study [93], confirm that mRNA vaccines seem to be well tolerated in patients with AIAIDs, without the induction of flare-ups or more frequent and serious AEs than in the general population. Recently, herpes zoster appearance was described in 6/491 (1.2%) patients with IMIDs (Immune-Mediated Inflammatory Disorders) following an mRNA vaccine [94]. In the general population, mRNA vaccines also show a very low frequency of serious AEs, lower than those observed in vectored vaccines. In general, patients with AIAIDs do not have contraindications to the COVID-19 vaccination. This remains true also for selective pathologies, such as vasculitis and/or autoimmune cytopenias, even in cases of previous neutropenia, anemia, and/or lymphocytopenia (the limits of the lymphocytopenia here considered are light < 1500/µL > 1000/µL; moderate < 1000/µL > 500/µL; severe < 500/µL [95,96,97]). The only risk is inducing a reduced and partially protective immune response. Patients should be vaccinated when the disease is stable in remission, in analogy with the behavior adopted with the traditional vaccines. Following the alarm caused by the appearance of VITT in rare subjects who had received the first dose of a viral vector non-replicating vaccine, such as Vaxzevria from AstraZeneca and Janssen/Johnson & Johnson, the experts have faced the issue of COVID-19 vaccine compatibility with primary or PIDs-associated thrombocytopenia, with primary or secondary anti-phospholipid syndrome, and with taking anti-coagulant therapy. Regarding thrombocytopenia, 20 cases were recently described in the USA following Pfizer and Moderna vaccines [51], only three of whom presented with pre-vaccine thrombocytopenia. Although the relationship to the vaccine has not been definitively demonstrated, in almost all cases (19/20) thrombocytopenia appeared after the first vaccine dose. Regarding the viral vector non-replicating vaccines (AstraZeneca and Janssen/Johnson & Johnson), the very rare, but unpredictable, VITT (with cerebral and/or splanchnic or diffuse thrombosis) is very severe with a bad prognosis [36], especially in women < 60 years old. Like the post-Pfizer and Moderna vaccine thrombocytopenia, which is not associated with thrombosis and has a better prognosis, VITT generally appears in apparently healthy individuals without pre-vaccine thrombocytopenia. Thus, subjects with primary or PIDs-associated thrombocytopenia must be immunized against COVID-19; however, like the non-thrombocytopenic patients with AIAIDs, PIDs, and SIDs, they should only be vaccinated with mRNA vaccines, and should not receive viral vector non-replicating vaccines. The same recommendation is true for patients with primary or secondary anti-phospholipid syndrome, who must be immunized only during remission. In addition, both types of patients (the thrombocytopenic ones and those with anti-phospholipid syndrome) should be carefully monitored in the post-vaccine phase. Finally, patients under anti-coagulation therapy, such as heparin, should not discontinue the therapy if mRNA vaccine administration is planned. Even though there is no evidence that heparin may precipitate VITT, the similar pathogenesis of heparin-induced thrombocytopenia (e.g., appearance of anti-PF4 antibodies) makes it seem prudent to replace heparin-like with non- heparin-like anticoagulants if administration of a viral vector non-replicating vaccine is planned [34].

Even patients with PIDs and SIDs do not seem to have contraindications to COVID-19 vaccines. In fact, no higher frequency of AEs was observed in two preliminary studies, the first one on 11 immune deficient patients (10 PIDs and 1 SID) [98] and the other on 26 patients with inborn errors of immunity (26 PIDs) [99], where substantial safety of the mRNA Pfizer vaccine and satisfactory immunogenicity was observed, except for the four patients with X-linked agammaglobulinemia, in whom an adaptive cellular response was observed. A recent study confirmed the lack of antibody response in patients with X-linked agammaglobulinemia, compensated by the induction of an adaptive cellular response, whereas the response of patients with Common Variable Immunodeficiency was found to be unsatisfactory and non-protective at both cellular and humoral levels [100]. Safety and substantial immunogenicity were observed in patients with onco-hematological diseases [101,102] and in hemodialysis patients [103]. Despite the absence of comparative studies between mRNA and viral vector non-replicating vaccines, data from scientific studies and reports of regulatory authorities indicate better protection from mRNA vaccines. Regarding safety, viral vector non-replicating vaccines have shown a higher frequency of severe AEs, such as the highly lethal VITT. Moreover, preliminary studies [48,49,50] have shown that mRNA vaccines may be safely administered to patients with AIAIDs. Thus, based on the current state of knowledge, the Task Force considered that mRNA vaccines should be the only ones to be administered in patients with AIAIDs, PIDs, and SIDs, unless more convincing safety and efficacy data on the use of viral vector non-replicating vaccines becomes available.

## 4. Immunosuppressive/Immunomodulating Therapy and COVID-19 Vaccines

**Figure 1:**
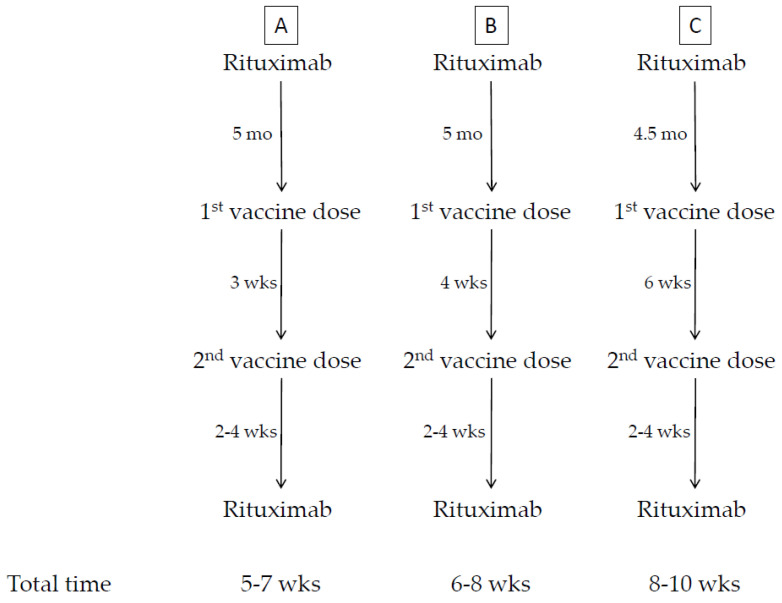
Proposed protocol of time intervals between ongoing therapy with rituximab and anti-COVID-19 vaccination. The time intervals between two consequent treatments with rituximab and anti-COVID-19 vaccination are provided. Total time indicates the duration of the postponement of the next scheduled cycle of rituximab since the first vaccination. (**A**) Vaccination with BNT162 vaccine (Comirnaty, Pfizer) following the time interval between the two doses as approved in the label. (**B**) Vaccination with mRNA-1273 vaccine (Spikevax, Moderna) following the time interval between the two doses as approved in the label. (**C**) Vaccination with either BNT162 vaccine (Comirnaty, Pfizer) or mRNA-1273 vaccine (Spikevax, Moderna) with delay between the two doses, as implemented in Italy. Abbreviations: mo, months; wks, weeks.

The vaccine is more effective the lower the immunosuppression; however, the risk of a flare-up of the underlying disease following interruption of an immunosuppressive drug is real, thus, in general, no modification of immunosuppressive therapy, prior to, during, or following vaccination, is advisable. However, based on what has been observed with traditional vaccines (Table 1, Table 2 and Table 3) [1,2,3,4,5,6,7,8,9,10,11,12,13,14,15], immunosuppressive drugs may negatively interfere with the immune response to vaccines. There is agreement between the European League against Rheumatism (EULAR) [104] and the American College of Rheumatology (ACR) [105] that treatment with corticosteroids (CCS) and anti-CD20 monoclonal antibodies, such as rituximab as an inducer of B cell death, markedly reduce the antibody response. Treatment with CCS should, therefore, be reduced to the minimum level compatible with disease control, which, however, is different between ACR (<20 mg/day of prednisone-equivalent) and EULAR (<10 mg/day). Even anti-CD20 monoclonal antibodies (mAbs) must be administered far away from vaccination (if clinical conditions allow, vaccination should be administered four weeks before the next scheduled cycle of rituximab and this cycle should be delayed and administered two–four weeks following the second vaccine dose). Recently, the issue of delaying the second dose of mRNA vaccines has been discussed and implemented in many countries, including Italy. Such a delay may be as long as two–three weeks more than the period approved in the label, up to a total of six weeks. In this case, the specialist may be forced to anticipate vaccination by two weeks, thus starting vaccination eight, instead of four, weeks before the next scheduled cycle of rituximab. A simplified scheme about the time intervals between ongoing therapy with rituximab and anti-COVID-19 vaccination is provided in Figure 1.

For the other immunosuppressive drugs, the EULAR and ACR positions are quite divergent. In fact, EULAR does not consider further possible interruptions of the immunosuppressive treatment, whereas ACR recommends the possible suspension of methotrexate (MTX), abatacept, and Janus kinase inhibitors (JKI), provided that the clinical conditions allow. MTX should be interrupted for one week after each vaccine dose, and subcutaneous abatacept for one week before and after the first vaccine dose. For intravenous abatacept, the first vaccine dose should be administered, if possible, four weeks following the drug infusion and the next infusion should be delayed by one week, if clinical conditions allow. JKI should be interrupted for one week after each vaccine dose. The last update of the ACR guidance was even more restrictive by introducing discontinuation of mycophenolate for one week following each vaccine dose and of acetaminophen and non-steroidal anti-inflammatory drugs (NSAIDs) 24 h prior to vaccination [106]. However, the limits on the use of immunosuppressive drugs in the vaccination period have been based on very few studies on traditional vaccines, generally counterbalanced by a higher number of reports, which did not show any significant negative interference in the immune response to vaccines, thus providing an explanation of the divergent recommendations. Recently, some studies have been published on the immune response to two-dose anti-COVID-19 mRNA vaccines in patients with AIAIDs under immunosuppressive therapy [49,50,93,107,108,109]. Geisen et al. reported that the immune response was adequate in 26 patients with chronic inflammatory conditions under immunosuppressive therapy [49]; Simon et al. studied 84 patients with AIAIDs and 10% of the patients were unable to respond vs. 1% of the healthy controls; moreover, such a reduced response was ascribed to the disease itself and not to the immunosuppressive therapy [50]. Spiera et al. analyzed 83 patients with rheumatic diseases, 30 of whom were treated with rituximab, and did not observe any immune response in 20/30 patients under rituximab, and in only one patient under belimumab [107]. Ruddy et al. observed a generally good response in 404 patients with AIAIDs, but a reduced response in patients under mycophenolate, rituximab, and CCS [108]. Haberman et al., by studying 82 patients with AIAIDs, observed a reduced antibody response (62.2% vs. over 90%) and a reduced CD8+ cellular response in the 45 patients under MTX [109]. Finally, Furer et al., in the largest multicentric study carried out in Israel on 686 patients immunized with two doses of Comirnaty Pfizer vaccine, observed a severely reduced immune response in patients under rituximab and a moderately reduced immune response in patients on CCS, abatacept, and mycophenolate mofetil, whereas only a mild impairment of immune response was observed because of MTX [93]. Thus, (even with mRNA vaccines) analogous to what has been observed with traditional vaccines, patients with AIAIDs may present a reduced vaccine-induced immune response because of the disease itself as well as immunosuppressive therapy, with CCS and anti-CD20 mAbs nearly always implicated in the immunosuppressive effect. However, for anti-CD20 mAbs, the marked reduction of the antibody response is associated with substantial maintenance of the adaptive cellular immunity, as originally observed with the influenza vaccine [13] and recently confirmed with anti-COVID-19 vaccines [110], even towards some variants of concern, such as B.1.1.7 and B.1.351 [111]. All the authorized COVID-19 vaccines can stimulate specific cellular immunity (Table 4) Moreover, immunosuppressive treatment has even been associated in some studies, with a protective effect on the cytokine release syndrome [112] observed in some cases of SARS-CoV-2 infection. All these considerations may explain the different evaluations by the different Scientific Societies/Colleges, including EULAR [104], ACR [105,106], and the Korean College of Rheumatology [113], which has an intermediate position compared to the other two. All agree on the inhibitory effect of CCS and anti-CD20 mAbs [114], either isolated or in combination. It should be underlined that in patients with stabilized AIAIDs, the dosage of the immunosuppressive drugs is generally lower than the threshold considered high-level [4]. Therefore, in these patients, it is preferable to provide partial protection with vaccination rather than risk disease reactivation because of immunosuppressive therapy interruption. CCS should be reduced up to the lowest level still compatible with disease control, at any rate <10 mg/day of prednisone-equivalent, and the anti-CD20 mAbs should be appropriately spaced around the vaccinations according to the ACR recommendations. For MTX, abatacept, JKI, and mycophenolate, the decision should be made by the immune system specialist based on the severity of the clinical picture and the possible risk of the even temporary interruption of the immunosuppressive treatment. The Task Force position regarding vaccination and immunosuppressive therapy is summarized in Table 6

Regarding SIDs, the rate of response by patients with solid tumors is optimal and higher than the one by patients with hematologic malignancies (98% vs. 85%, respectively, especially in case of anti-CD20 therapy (70%) and stem cell transplantation (73%) [100], thus underlining once more the negative influence of some immunosuppressive treatments and the recommendation, when feasible, to vaccinate before immunosuppressive treatment is started.

## 5. Should Patients with AIAIDs, PIDs, and SIDs Be Prioritized in the Access to Vaccines?

There is no general agreement regarding priority criteria for anti-COVID vaccine access for patients with AIAIDs, PIDs, and SIDs because there is no consensus on their actual risk of infection or developing a more serious disease. A large meta-analysis on over 300,000 patients with AIAIDs has shown that the risk of getting a COVID-19 infection is significantly higher than that of the general population, especially associated with previous steroid intake. However, COVID-19 prognosis does not seem to be any worse than that of the general population [117], even though single studies report a higher disease severity [118].

PIDs [119,120,121,122] do not show a higher risk of getting COVID-19 infection or of increased lethality compared to the general population, except for rare forms of congenital defects of interferon production, whereas conversely, SIDs, especially onco-hematological patients [102,123], are at higher risk of infection and lethality.

The best criterion, therefore, to evaluate the priority level of access to COVID-19 vaccine in these patients is the clinical one [124]: in patients with AIAIDs under heavy immunosuppressive therapy and a history of serious and recurrent infections, the immunology specialist will determine the priority level, in keeping with the *Raccomandazioni ad interim sui gruppi target della vaccinazione anti-SARS-CoV-2/COVID-19 (GURI 24.3.2021),* elaborated by the Italian Ministry of Health. It is necessary to emphasize that vulnerable patients with markedly dysfunctional immune systems may act as an incubator for SARS-CoV-2 variants. Because the established infection is not easily eliminated, it becomes chronic and the virus, under the pressure of the immune system, which is unable to clear it, mutates for survival [125]. This is a further consideration for prioritizing these patients in the access to vaccines to prevent the infection or, at least, a chronic infection. The Task Force does not consider it appropriate to interrupt/discontinue immunosuppressive therapy, excepting for high dosage CCS (≥10 mg of prednisone-equivalent [104]) and anti-CD20mAbs, such as rituximab [103,104,105] (Table 3). Moreover, in the case of MTX [105,106], abatacept [105,106], JKI [105,106], and mycophenolate [106], the immunology specialist should evaluate the possible discontinuation based on the clinical patients’ characteristics.

Clinical criteria should guide the management of patients with PIDs and SIDs as well. For these patients, vaccination of cohabiting relatives and healthcare providers is recommended.

## 6. Recommendations

1. Anti-COVID-19 mass vaccination has personal and social benefits. Preventing the disease in single individuals may allow easier achievement of herd immunity, which is needed to interrupt the viral spread and protect individuals who cannot be vaccinated.
2. Vaccinations are a safe and effective tool for prevention and control of infectious diseases. Patients with AIAIDs, PIDs, and SIDs are at higher risk of infections, including those by SARS-COV-2. Few studies have addressed the issue of anti-COVID vaccination in these patients, but many are available on the safety, immunogenicity, efficacy, and possible contraindications of traditional vaccines in AIAIDs, PIDs, and SIDs patients. These studies may represent the basis on which to recommend the anti-COVID-19 vaccines [1,2,3,4,5,6,7,8,9,10,11,12,13,14,15] (Table 1, Table 2 and Table 3)
3. The vaccine is more effective the lower the immunosuppression; however, the risk of a flare-up of the underlying disease after the interruption of an immunosuppressive drug is real, thus, in general, no modification of immunosuppressive therapy either during or following vaccination is advisable. In particular cases, according to the clinical picture and the drugs used, modifications and/or discontinuations of immunosuppressive therapy may be recommended by the immunology specialist.
4. In general, inactivated vaccines or vaccines containing non-infectious viral sequences may be safely administered to patients with AIAIDs, PIDs, and SIDs in clinical remission [1,2,3,4,5,6,7,8,9,10,11,12,13,14,15] (Table 1, Table 2 and Table 3). Anti-COVID-19 vaccines of the traditional type that are composed by inactivated virus [31,32,33] or recombinant Spike protein and adjuvant [19], for which wider scientific knowledge and clinical experience are available, could preferentially be indicated in patients with AIAIDs, PIDs, and SIDs; however, no traditional vaccines are approved by the European Medicines Agency (EMA) yet (Table 4).
5. Viral vector non-replicating anti-COVID-19 vaccines, such as the ones from AstraZeneca (Vaxzevria) [21,22], Janssen/Johnson & Johnson [23,24], Cansino Biological Inc. [25], and Gamaleya Research Institute [26,27] (Table 2), are known based on application to the anti-Ebola vaccine, however our knowledge of them is very limited. One problem with these vaccines is the previous and effective anti-viral vector immune response, which may totally inactivate the vaccine; however, this problem involves the general population, not only patients with AIAIDs, PIDs, and SIDs, and may be reduced by using viral vectors from primates. Although during the registration studies substantial safety of these vaccines has been observed [21], lack of patients with AIAIDs, PIDs, and SIDs in these studies does not allow definitive conclusions about the safety and efficacy of these vaccines in these patients. Moreover, recently the discovery of very rare, but severe and often lethal, cases of VITT prompted the regulatory agencies of many countries to substantially limit the use of these vaccines.
6. Anti-COVID-19 mRNA vaccines, such as those developed by Pfizer/Biontech [28] and Moderna [29], are brand new and the first ones to be used on a large scale. During the pre-approval studies, after four months of observation, the groups treated with the two vaccines showed an efficacy of 95% and 94.1%, respectively, compared to the group of subjects who had received placebo, and the safety was considered optimal (Table 4) [28,29]. The mRNA vaccine effectiveness has also been calculated at 91% and 81% after the complete vaccine cycle or only the first dose, respectively [126]. Although in the pre-approval studies details on the possible presence of patients with AIAIDs in the study population have not been provided, recent studies have reported substantial safety and immunogenicity of these vaccines in patients with AIAIDs [48,49,50,92,106,107,108]. Even in PIDs, two preliminary studies [98,99] have shown the substantial safety and immunogenicity of the mRNA vaccines. Finally, COVID-19 vaccines were safe and immunogenic in onco-hematologic pathologies, and mRNA vaccines were more immunogenic than the adenoviral vaccine [101]. Despite the absence of currently released definitive recommendations, the cited preliminary studies confirm that mRNA vaccines seem to be well tolerated in patients with AIAIDs, PIDs, and SIDs. In the general population as well, mRNA vaccines show a very low frequency of serious adverse events, lower than those observed in vectored vaccines. The Task Force believes that, based on the data reported above, mRNA vaccines should be chosen for use in clinically stabilized patients with AIAIDs, PIDs, and SIDs.
7. There is no general agreement regarding priority criteria for anti-COVID vaccine access for patients with AIAIDs, PIDs, and SIDs, because there is no consensus on their actual risk of infection or developing a more serious disease. The best criterion to evaluate the priority level of access to COVID-19 vaccine in these patients is the clinical one [124]: in patients with AIAIDs under heavy immunosuppressive therapy and a history of serious and recurrent infections, the immunology specialist should determine the priority level, in keeping with the *Raccomandazioni ad interim sui gruppi target della vaccinazione anti-SARS-CoV-2/COVID-19 (GURI 24.3.2021),* elaborated by the Italian Ministry of Health. Clinical criteria should guide the management of patients with PIDs and SIDs as well. For these patients, vaccination of cohabiting relatives and healthcare providers is recommended.
8. The Task Force does not consider it appropriate to interrupt/discontinue immunosuppressive therapy, excepting for high dosage CCS (≥10 mg of prednisone-equivalent [104]) and anti-CD20 mAbs, such as rituximab [103,104,105] (Table 6). Moreover, in the case of MTX [105,106], abatacept [105,106], JKI [105,106], and mycophenolate [106], the immunology specialist will evaluate the possible discontinuation based on the patients’ clinical characteristics. Regarding PIDs, the risk of non-response should be evaluated, especially in severe combined and humoral immunodeficiencies. In the case of non-response passive immunotherapy with mAbs or convalescent plasma, vaccination should carefully be considered.
9. The patient’s immunology specialist should be continuously updated and available for evaluating the vaccination risk level (disease activity and immunosuppression consequent to the current therapy) of the patient. The specialist should be continuously informed and able to provide all information useful for handling treatment during the vaccination period.
10. In keeping with the activity of the Italian Drug Agency (AIFA) to inform the whole population, the three Italian Immunological Societies will make available all data coming from studies carried out on immunized populations as soon as possible, using the sites or the traditional communication channels available to the members of scientific societies and the patient associations.

## 7. Conclusions

The COVID-19 pandemic has presented an unprecedented global challenge for humankind by causing deep and unimaginable consequences at the health, social, and economic levels. However, combined efforts in research have paved the way for an innovative new class of highly effective vaccines to be developed in less than one year. These vaccines, never used before, appear safe, effective, and easy to be tailored to new viral threats that may appear in the future. We believe that these new vaccines have a bright future and potentially represent powerful preventative weapons against dangerous pathological entities.

## Figures and Tables

**Table 1:** Inactivated and live traditional vaccines (not anti-COVID-19) in patients with AIAIDs and influence of the relative therapies.

AIAIDs	Corticosteroids	IVIg/scDMARDs	bDMARDs/tsDMARDs	Inactivated Vaccines	Live Vaccines
	High doses: ≥20 mg/day prednisone-equivalent for 1–2 weeks; non immunosuppressive doses: 7.5 mg/day	Sulphasalazine, hydroxichloroquine, azathioprine, mycophenolate, methotrexate, leflunomide, cyclosporine, tacrolimus, cyclophosphamide	infliximab, adalimumab, etanercept, golimumab, certolizumab, rituximab, tocilizumab, abatacept, anakinra, canakinumab, belimumab, secukinumab, ixekizumab, ustekinumab, tofacitinib, baricitinib	Hepatitis A/B, human papillomavirus, influenza, herpes zoster, inactivated poliovirus, pneumococcus, tetanus/diphtheria/pertussis, polysaccharide typhoid fever, Hib, meningococcus	Measles/Mumps/Rubella, Varicella, BCG, Ty21A, Yellow fever
Rheumatoid arthritis, Systemic lupus, erythematosus Sjögren Syndrome, Anti-phospholipid syndrome, Systemic sclerosis, Polymyositis/ dermatomyositis, Vasculitis, Psoriatic arthritis, Spondyloarthritis, Familiar Mediterranean Fever, Periodical fever syndromes, Type 1 diabetes, Inflammatory bowel diseases, Multiple sclerosis	At high doses, they seem to interfere with the immune response to vaccines. Combination steroids/anti-TNF-α is particularly associated with the infection risk [10].	IVIg should not be administered together with live vaccines due to the risk of vaccine inactivation [11], or with an inactivated vaccine because evaluating vaccine immunogenicity become simpossible. csDAMRDs are generally well tolerated at the doses generally used in IMIDs and they do not seem to interfere with the immune response. For methotrexate, a negative interference with the pneumococcal vaccine has been described, which has not been confirmed with the conjugate vaccine [12].	When used alone, they are well tolerated and do not induce immunosuppression; immunosuppression is induced when they are used in combination. Abatacept and tofacitinib have been associated with a slightly reduced response to influenza and pneumococcus vaccines. Rituximab markedly reduces antibody response, but it does not seem to modify the adaptive cellular one [13]. Vaccination should be carried out before starting therapy with rituximab; in case of impossibility, it should be carried out 6 months after the last infusion of rituximab and 1 month before the next one.	Generally allowed, influenza and pneumococcus recommended, and, in particular subjects, hepatitis B, papillomavirus and herpes zoster.	Generally contraindicated, Measles/Mumps/Rubella seems well tolerated. Caution for Yellow fever, even though a recent review seems to partially reduce these fears [14].

AIAIDs = Autoimmune-autoinflammatory disorders; IMIDs = Immune-Mediated Inflammatory Disorder(s); IVIg = Intravenous immunoglobulin; scDMARDs = Synthetic conventional Disease-Modifying Anti-Rheumatic Drugs; bDMARDS = Biologic Disease-Modifying Anti-Rheumatic Drugs; tsDMARDs = Targeted synthetic Disease-Modifying Anti-Rheumatic Drugs; Hib = *Haemophilus influenzae* b, BCG = Bacillus Calmette-Guérin; Ty21A = oral live vaccine for typhoid fever.

**Table 2:** Inactivated and live traditional vaccines (not anti-COVID-19) in patients with PIDs and influence of the relative therapies.

PIDs	Corticosteroids	IVIg/SCIg	HSCT/Gene therapy	Inactivated Vaccines	Live Vaccines
	High doses: ≥20 mg/day prednisone-equivalent for 1–2 weeks; non immunosuppressive doses: 7.5 mg/day			Hepatitis A/B, human papillomavirus, influenza, herpes zoster, inactivated poliovirus, pneumococcus, tetanus/diphtheria/pertussis, polysaccharide typhoid fever, Hib, meningococcus,	Measles/Mumps/Rubella, Varicella, BCG, Ty21A, Yellow fever
Major antibody defects (XLA, CVID) Minor antibody defects (Defect of: IgA, IgG subclasses, specific antibodies) SCID—CID MSMD Invasive bacterial infections CMCD Defects of TLR Defects of IL12/IFN-ɣ pathway Defects of complement Congenital phagocyte defects Complete DiGeorge Syndrome Partial DiGeorge Syndrome Ataxia-Telangiectasia Wiskott-Aldrich Syndrome Hyper-IgE Syndrome IPEX Syndrome APECED Syndrome	At high doses, they seem to interfere with the immune response to vaccines.	IVIg should not be administered together with live vaccines due to the risk of vaccine inactivation [11], or with an inactivated vaccine because evaluating vaccine immunogenicity becomes impossible.	After 1 year following engraftment and lack of GVHD, it is possible to set the vaccination schedule with inactivated vaccines. Live vaccines should not be set before 2 years from transplant [15].	Generally allowed in all the PIDs, excepting SCID and complete DiGeorge syndrome, in which only the polysaccharide vaccines (meningococcus, pneumococcus, Hib) are allowed.	Generally contraindicated, MMR and Varicella seem well tolerated in the minor antibody defects, complement defects, congenital phagocyte defects, partial DiGeorge syndrome, ataxia-telangiectasia, and hyper-IgE syndrome. Caution should be used for Yellow fever vaccine.

PIDs = Primary immunodeficiencies; IVIg = Intravenous immunoglobulin; SCIg = Subcutaneous immunoglobulin BCG = Bacillus Calmette-Guérin; Ty21A = oral live vaccine for typhoid fever; HSCT = Hematopoietic Stem-Cell Transplantation; XLA = X-linked agammaglobulinemia; CVID = Common variable immunodeficiency; SCID = Severe combined immunodeficiency; CID = Combined immunodeficiency; MSMD = Mendelian susceptibility to mycobacterial disease; CMCD = Chronic mucocutaneous candidiasis disease; TLR = Toll-like receptors; GVHD = Graft versus host disease; Hib = *Haemophilus influenzae* type b; IPEX = Immune dysregulation, poly-endocrinopathy, enteropathy X-linked; APECED = Autoimmune poly-endocrinopathy-candidiasis-ectoderma-dystrophy.

**Table 3:** Inactivated and live traditional vaccines (not anti-COVID-19) in patients with SIDs and influence of the relative therapies.

SIDs	Corticosteroids	IVIg/SCIg	Chemotherapy/Biologics/Janus Kinase Inhibitors	Inactivated Vaccines	Live Vaccines
	High doses: ≥20 mg/day prednisone-equivalent for 1–2 weeks; non immunosuppressive doses: 7.5 mg/day			Hepatitis A/B, human papillomavirus, influenza, herpes zoster, inactivated poliovirus, pneumococcus, tetanus/diphtheria/pertussis, polysaccharide typhoid fever, Hib, meningococcus	Measles/Mumps/Rubella, Varicella, BCG, Ty21A, Yellow fever
Transplanted patients Hematological patients Oncological patients Patients with IMIDs on immunosuppression Dialysis patientsSevere asthma/COPD Splenectomized patients HIV-infected patients	At high doses they seem to interfere with the immune response to vaccines.	IVIg should not be administered together with live vaccines due to the risk of vaccine inactivation [11], or with an inactivated vaccine because evaluating vaccine immunogenicity becomes impossible.	In case of high-level immunosuppression by chemotherapy in onco-hematological pathologies, inactivated vaccines should preferably be administered either before or after, but not during, the treatment.	Generally allowed in all the reported SIDs. Polysaccharide vaccines (pneumococcus, meningococcus, Hib) are specifically recommended in the splenectomized patients. Polysaccharide and influenza vaccines are recommended in transplanted patients and in the other SIDs. Hepatitis A and B in liver transplanted patients. Hepatitis B in HIV-infected patients. Two months after the transplant it is possible to plan vaccinations with inactivated vaccines [7]. In Rituximab-treated patients, vaccination should be carried out at least 6 months after the last infusion.	Generally contraindicated, Measles/Mumps/Rubella and Varicella are allowed in HIV-infected patients, provided that they have CD4 ≥ 200/µL. Caution for Yellow fever, even though a recent review seems to partially reduce these fears [14].

SIDs = Secondary immunodeficiencies; IMIDs = Immune-Mediated Inflammatory Disorder(s); IVIg = Intravenous immunoglobulin; SCIg = Subcutaneous immunoglobulin; Hib = *Haemophilus influenzae* type b; BCG = Bacillus Calmette-Guérin; Ty21A = oral live vaccine for typhoid fever.

**Table 5:** SARS-CoV-2 variants of concern (from Ref. [64] modified).

WHO Label	Lineage	Country of Isolation	Transmissibility	Disease Severity	Vaccine Protection
Alpha	B.1.1.7	United Kingdom	Increased	Increased	Unmodified
Beta	B.1.351	South Africa	Increased	Increased	Reduced
Gamma	P.1	Brazil	Increased	Increased	Reduced
Delta	B.1.617.2	India	Increased	Increased	Reduced

**Table 6:** Guide to using anti-COVID-19 vaccination in patients with AIAIDs under immunosuppressive treatment [from Ref. [103] modified].

Drug	Modification of Therapy	Modification of Therapy in Relation to Vaccination
Hydroxychloroquine	NO	
Apremilast	NO	
IVIG	NO	
Glucocorticoids (Prednisone-equivalent < 10 mg/day)	NO	
Glucocorticoids (Prednisone-equivalent ≥ 10 mg/day)		The dose should be reduced to <10 mg/die, if possible, before each vaccine dose
Sulphasalazine	NO	
Leflunomide	NO	
Mycophenolate mofetil		Delay the dose, if >20 mg/week, for 1 week for each vaccine dose, in case of stable disease *
Azathioprine	NO	
Cyclophosphamide (Oral)	NO	
TNFα inhibitors (Adalimumab, Infliximab, Golimumab, Certolizumab, Etanercept)	NO	
Anti-IL-6R moAb (Tocilizumab)	NO	
IL-1β inhibitors (Anakinra, Canakinumab)	NO	
Anti-IL-17A moAbs (Secukinumab, Ixekizumab)	NO	
Anti-IL-12/23 moAb (Ustekinumab)	NO	
Anti-IL-23 moAbs (Tildrakizumab, Guselkumab, Risankizumab)	NO	
Anti-Blys moAb (Belimumab)	NO	
Calcineurin inhibitors (oral)	NO	
Methotrexate		Delay the dose, if >20 mg/week, for 1 week for each vaccine dose, in case of stable disease *
JAK inhibitors		Delay the dose for 1 week for each vaccine dose *
Abatacept sc		Temporary interruption 1 week before and 1 week after each vaccine dose *
Abatacept iv		Each vaccine dose should be administered 4 weeks after the last infusion and the next infusion should be postponed 1 week *
Cyclophosphamide iv		The infusion should be administered 1 week after vaccine *
Anti-CD20 moAb (Rituximab)		The first vaccine dose should be administered 4 weeks before the next scheduled cycle. Rituximab may be administered not before 3 weeks after the second vaccine dose, provided that the patient’s clinical condition allows.

* The risk-benefit evaluation of temporary immunosuppressive treatment interruption should be done by the immunology specialist based on the clinical picture. Regarding PIDs, the presence of Ig replacement therapy does not seem to have interfered with the cellular and humoral immune response observed in most patients, except for patients with X-linked agammaglobulinemia, who did not show antibody response, but were protected by the adaptive cellular immunity [98,99]. However, immunoglobulin for intravenous (IVIg) or subcutaneous (SCIg) use should not be administered in the same time period as vaccination, because it is impossible to check the humoral vaccine response, given that IVIg/SCIg may contain different concentrations of anti-SARS-CoV-2 antibodies [115,116]. Considering that the threshold level for protection is still unknown, it is generally recommended to avoid pre- and post-vaccine anti-SARS-CoV-2 antibody testing. Nevertheless, post-vaccine antibody monitoring is indicated in vulnerable immunosuppressed patients [75,76], especially in patients with severe combined and humoral immune deficiency. In fact, in the case of lack of cellular and antibody response, in immunosuppressed patients the strategy of additional boosters may be tried, whereas in PIDs passive immunotherapy with mAbs or convalescent plasma may be considered should the anti-SARS-CoV-2 antibody levels in the IVIg/SCIg used for replacement therapy not be adequately represented.
